# Quantitative Consequences of Protein Carriers in Immunopeptidomics and Tyrosine Phosphorylation MS^2^ Analyses

**DOI:** 10.1016/j.mcpro.2021.100104

**Published:** 2021-05-28

**Authors:** Lauren E. Stopfer, Jason E. Conage-Pough, Forest M. White

**Affiliations:** Department of Biological Engineering, Koch Institute for Integrative Cancer Research, Center for Precision Cancer Medicine, Massachusetts Institute of Technology, Cambridge, Massachusetts, USA

**Keywords:** phosphotyrosine, phosphoproteomics, immunopeptidomics, MHC class I, protein carrier, BOOST channel, quantification, AGC, automatic gain control, BCA, bicinchoninic acid assay, CDK4/6, cyclin-dependent kinase 4/6, CV, coefficient of variation, DDA, data-dependent acquisition, DMSO, dimethyl sulfoxide, EGF, epidermal growth factor, EGFR, epidermal growth factor receptor, FAIMS, high-field asymmetric waveform ion mobility spectrometry, HCD, higher-energy collision dissociation, HipMHC, heavy-isotope labeled pMHCs, IFN-γ, interferon-gamma, IT, injection time, MS, mass spectrometry, MS3, triple-stage mass spectrometry, nCE, normalized collision energy, PBS, phosphate buffered saline, PC, principal component, PCA, principal component analysis, PD, proteome discoverer, pMHC, peptide major histocompatibility complex, PSM, peptide spectrum match, pTyr, tyrosine phosphorylation, PV, pervanadate, RT, room temperature, SCP, single-cell proteomics, SP3, single-pot solid-phase-enhanced sample preparation, TMT, tandem mass tags

## Abstract

Utilizing a protein carrier in combination with isobaric labeling to “boost” the signal of other low-level samples in multiplexed analyses has emerged as an attractive strategy to enhance data quantity while minimizing protein input in mass spectrometry analyses. Recent applications of this approach include pMHC profiling and tyrosine phosphoproteomics, two applications that are often limited by large sample requirements. While including a protein carrier has been shown to increase the number of identifiable peptides in both applications, the impact of a protein carrier on quantitative accuracy remains to be thoroughly explored, particularly in relevant biological contexts where samples exhibit dynamic changes in abundance across peptides. Here, we describe two sets of analyses comparing MS^2^-based quantitation using a 20× protein carrier in pMHC analyses and a high (~100×) and low (~9×) protein carrier in pTyr analyses, using CDK4/6 inhibitors and EGF stimulation to drive dynamic changes in the immunopeptidome and phosphoproteome, respectively. In both applications, inclusion of a protein carrier resulted in an increased number of MHC peptide or phosphopeptide identifications, as expected. At the same time, quantitative accuracy was adversely affected by the presence of the protein carrier, altering interpretation of the underlying biological response to perturbation. Moreover, for tyrosine phosphoproteomics, the presence of high levels of protein carrier led to a large number of missing values for endogenous phosphopeptides, leading to fewer quantifiable peptides relative to the “no-boost” condition. These data highlight the unique limitations and future experimental considerations for both analysis types and provide a framework for assessing quantitative accuracy in protein carrier experiments moving forward.

## Highlights

• A carrier proteome may enhance detection of low abundance pMHC and pTyr peptides.
• A carrier boosted IDs in pMHC analyses; quantitation suffered from ratio compression.
• A 9× carrier provided higher quantitative accuracy over a 100× carrier in pTyr analyses but offered minimal benefit in data quantity.
• Experimental design should be rigorously evaluated to ensure quantitative accuracy.

## Graphical Abstract

**Figure:**
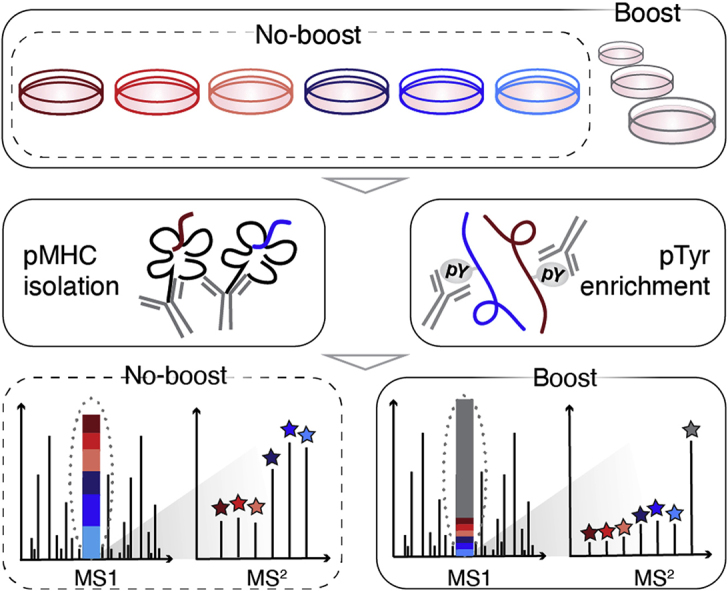

## In Brief

Use of a protein carrier offers the opportunity to increase data quantity while minimizing sample input requirements, an attractive strategy for tyrosine phosphorylation and immunopeptidomics analyses. Still, the quantitative consequences of including a protein carrier in pMHC and pTyr analyses, particularly in a biological context, remain poorly understood. Here, a comparison of experiments with and without a carrier proteome reveals critical limitations, suggesting that experimental design of carrier experiments should be carefully assessed to avoid misinterpretation of data.

Mass spectrometry (MS)-based proteomics has historically been limited to analyzing bulk cell populations, largely due to losses during sample processing and limited instrument sensitivity. In recent years, several platforms have achieved protein expression profiling in single cells (*e.g.*, single-cell proteomics (SCP)), a notable advancement in proteomics. To overcome sensitivity limitations and acquire deep proteomics datasets, the majority of these platforms rely on isobaric labeling (*i.e.*, tandem mass tags (TMT)) for sample multiplexing and a signal “boosting” sample, or “carrier proteome.” (1, 2, 3) Carrier proteomes that have been utilized thus far contain a larger amount of protein than the noncarrier samples (1, 2, 3), an equivalent amount of protein but with a perturbation to increase the signal of interest (4), or both (5). Because all isobaric labels have an identical intact mass, the inclusion of a carrier proteome increases the precursor ion intensity, enabling enhanced detection of low-input or low-level samples.

Use of a carrier proteome has also recently been applied to peptide major histocompatibility complex (pMHC) profiling (*e.g.*, immunopeptidomics) and tyrosine phosphorylation (pTyr) analyses, both of which historically have required large sample inputs for sufficient signal detection by MS. For example, recent advances in pMHC profiling methods have decreased sample input requirements from >10^9^ cells to ~10^7^ cells, yet even this lower boundary still represents a major limitation in the clinical translatability of the approach (6, 7). Clinical specimens, including fine needle biopsies, typically do not provide enough material for deep pMHC profiling, and neoantigens are challenging to identify by MS, even with large sample quantities (8). Similarly, profiling pTyr peptides is possible using several hundred micrograms of input protein per channel in a multiplexed analysis (9), but there is continued effort to reduce sample requirements to enable pTyr profiling of fine needle biopsies, tissue sections, or even single cells.

Inclusion of a protein carrier has resulted in an increased number of identifiable peptides in multiplexed immunopeptidomics analyses as well as multiplexed phosphotyrosine analyses. Ramarathinam *et al.* utilized increased protein material, cellular or patient-derived xenograft tumors, as a protein carrier in class I pMHC experiments, while Fang *et al.* used tenfold higher cellular input of the control sample as a protein carrier, and Chua *et al.* used a protein carrier that had been treated with pervanadate (PV) to halt tyrosine phosphatase activity and thereby increase tyrosine phosphorylation levels (5, 10, 11). While these initial results are encouraging, the quantitative impact of boosting in both approaches remains poorly understood. Specifically, a carrier proteome may limit the instrument’s dynamic range, leading to reporter ion ratio compression and increase the number of missing values, thereby reducing data quality and/or data quantity, potentially altering biological interpretation (12).

Several studies have begun to address these critical questions, albeit with limitations. For instance, experiments to assess ratio suppression typically evaluate whether constant ratios of protein input material are preserved in the presence of a protein carrier, which is not reflective of many biological systems where subtler changes in a subset of peptides demonstrate altered quantitation against a background of unchanging signal (5, 12). Studies have also evaluated whether principal component analysis (PCA) can resolve differences between two cell populations in the presence of various protein carrier-to-signal ratios. However, these experiments generally use distinct cell types or cell lines, which have higher heterogeneity in peptide quantitation (1, 5, 12, 13).

Here, we describe results from analyses comparing MS^2^-based quantitation with and without the inclusion of a 20× protein carrier in pMHC analyses and a high (~100×) or low (~9×) carrier in pTyr analyses, using samples where fraction of peptides exhibit quantitative changes in signal. We estimated ratio compression in pMHC analyses using titrated isotopically labeled pMHCs and treated cells with a cyclin-dependent kinase 4/6 (CDK4/6) inhibitor to shift a subset of the pMHC repertoire in pathways related to cell cycle control (7). In pTyr experiments, epidermal growth factor (EGF) stimulation was used to drive a temporal pTyr response in a subset of the tyrosine phosphoproteome (14). In both applications, protein carriers altered peptide quantitation compared with the control experiment, inhibiting our ability to accurately interpret the biology underlying the cellular perturbations. Using these data, we define existing limitations for MS^2^-based analyses using protein carriers and highlight areas for future exploration that may enhance data quality through altered experimental design or acquisition framework.

## Experimental Procedures

### Cell Lines

SKMEL5 and A549 cell lines were obtained from ATCC (ATCC HTB-70 and CCL-185, respectively). Cells were maintained in DMEM medium (Corning) supplemented with 10% FBS (Gibco) and 1% penicillin/streptomycin (Gibco). Cells were routinely tested for *mycoplasma* contamination and maintained in 37 °C, 5% CO_2_. Experiments were performed on passages 4 to 8.

### Synthetic Peptide Standards

Heavy leucine-containing peptides were synthesized at the MIT Biopolymers and Proteomics Lab using standard Fmoc chemistry using an Intavis model MultiPep peptide synthesizer with HATU activation and 5 μmol chemistry cycles. Starting resin used was Fmoc-Amide Resin (Applied Biosystems). Cleavage from resin and simultaneous amino acid side chain deprotection was accomplished using: trifluoroacetic acid (81.5% v/v); phenol (5% v/v); water (5% v/v); thioanisole (5% v/v); 1,2-ethanedithiol (2.5% v/v); 1% triisopropylsilane for 1.5 h. Fmoc-Leu (^13^C_6_, ^15^N) was obtained from Cambridge Isotope Laboratories, and standard Fmoc amino acids were from NovaBiochem.

Peptides were subjected to quality control by MS and reverse-phase chromatography using a Bruker MicroFlex MALDI-TOF and Agilent model 1100 HPLC system with a Vydac C18 column [300 Å, 5 micron, 2.1 × 150 mm] at 300 μl/min monitoring at 210 and 280 nm with a trifluoroacetic acid/H_2_O/MeCN mobile-phase survey gradient.

### UV-mediated Peptide Exchange for hipMHCs

UV-mediated peptide exchange to generate hipMHCs was performed using recombinant, biotinylated Flex-T HLA-A∗02:01 monomers (BioLegend), using a modified version of the commercial protocol as previously described (7). Concentration of stable complexes following peptide exchange was quantified using the Flex-T HLA class I ELISA assay (BioLegend) as per the manufacturer’s instructions. ELISA results were acquired using a Tecan plate reader Infinite 200 with Tecan icontrol version 1.7.1.12.

### Peptide MHC Isolation and TMT Labeling

Cells were seeded in 10 cm plates and treated the following day for 72 h with DMSO control, 10 μM palbociclib (Selleckchem, PD-0332991), or 10 ng ml^−1^ human recombinant IFN-γ (ProSpec Bio). During harvest, cells were washed with 1× PBS and lifted with 0.05% Trypsin-EDTA (Gibco). Cells were pelleted, washed with 1× PBS, pelleted again, and resuspended in lysis buffer [20 nM Tris-HCl pH 8.0, 150 mM NaCl, 0.2 mM PMSF, 1% CHAPS, and 1× HALT Protease/Phosphatase Inhibitor Cocktail (Thermo Fisher)], followed by brief sonication to disrupt cell membranes. Lysate was cleared by centrifugation and quantified using bicinchoninic acid (BCA) protein assay kit (Pierce).

Peptide MHCs were isolated by immunoprecipitation (IP) as previously described (7). Briefly, per 1E7 cells, 200 μg of pan-specific anti-human MHC Class I (HLA-A, HLA-B, HLA-C) antibody (clone W6/32, Bio X Cell) was bound to 10 μl FastFlow Protein A Sepharose bead slurry (GE Healthcare) for 3 h rotating at 4 °C. Beads were washed 2× with IP buffer (20 nM Tris-HCl pH 8.0, 150 mM NaCl), after which cell lysate and hipMHCs were added and incubated rotating overnight at 4 °C. Beads were washed with 1× TBS and water, and pMHCs eluted in 10% formic acid for 20 min at RT. Peptides were isolated from antibody and MHC molecules using a passivated 10K molecule weight cutoff filter (PALL Life Science), lyophilized, and stored at −80 °C prior to TMT labeling.

To label pMHCs, 50 μg of pre-aliquoted Tandem Mass Tag 6-plex (TMT-6, Thermo Scientific) was resuspended in 20 μl anhydrous acetonitrile and lyophilized peptides were resuspended in 66 μl 150 mM triethylammonium bicarbonate, 50% ethanol. TMT/peptide mixtures were incubated on a shaker for 1 h at RT, and reactions were quenched with 0.3% of hydroxylamine. Samples were next combined and centrifuged to dryness. Sample cleanup was subsequently performed using SP3, as previously described (7, 15).

### pTyr Sample Preparation

A549 cells were seeded in 10 cm plates and serum depleted for 72 h prior to analysis. In EGF stimulation experiments, cells were stimulated with 5 EGF (PeproTech), flash frozen in liquid nitrogen, and lysed in 8M urea. PV-treated cells were incubated for 30 min with 30 μM PV at 37 °C prepared using 200 mM sodium orthovanadate, 1× PBS, and 30% hydrogen peroxide, followed by a 15 min incubation at RT protected from light. Cells were subsequently washed 1× with ice cold 1× PBS and lysed in 8 M urea.

Lysates were cleared by centrifugation at 5000*g* for 5 min at 4 °C, and protein concentration was measured by BCA (Pierce). Proteins were reduced with 10 mM DTT for 30 min at 56 °C, alkylated with 55 mM iodoacetamide for 45 min at RT protected from light, and diluted fourfold with 100 mM ammonium acetate, pH 8.9. Proteins were digested with sequencing grade modified trypsin (Promega) at an enzyme to substrate ratio of 1:50 overnight at RT. Enzymatic activity was quenched by acidifying with glacial acetic acid to 10% of the final solution volume, and peptides were desalted using C18 solid-phase extraction cartridges (Sep-Pak Plus Short, Waters). Peptides were eluted with aqueous 40% acetonitrile in 0.1% acetic acid and dried using vacuum centrifugation. Peptide concentration was measured by BCA to account for variation in sample processing, and peptides were subsequently lyophilized.

Lyophilized peptides were labeled with TMT-10plex in ~35 mM HEPES and ~30% acetonitrile at pH 8.5 for 1 h at room temperature. Hundred micrograms of peptide aliquots utilized 400 μg TMT, 900 μg^−1^ mg aliquots used 1600 μg TMT. Labeling reactions were quenched with 0.3% of hydroxylamine, and samples were pooled, dried by vacuum centrifugation, and stored at −80 °C prior to analysis.

Labeled peptide aliquots were resuspended in 400 μl of IP buffer [100 mM Tris-HCl, 0.3% NP-40, pH 7.4] and incubated with 60 μl protein G agarose bead slurry (Calbiochem) conjugated to an antibody cocktail containing 24 μg 4G10 (Millipore) and 12 μg PT66 (Sigma), rotating overnight at 4 °C. Beads were washed 1× with IP buffer, 3× with 100 mM Tri-HCl, pH 7.4, and eluted in two rounds of 25 μl 0.2% TFA. Phosphopeptides were further enriched using High-Select Fe-NTA Phosphopeptide Enrichment Kit (Thermo Scientific) following manufacturer’s instructions with minor adjustments as previously described (16). Peptide elutions were dried down using vacuum centrifugation to <2 μl total volume and resuspended in 5% acetonitrile in 0.1% formic acid for a total volume of 10 μl.

### MHC MS Data Acquisition

pMHC samples were analyzed using an Orbitrap Exploris 480 mass spectrometer (Thermo Scientific) coupled with an UltiMate 3000 RSLC Nano LC system (Dionex), Nanospray Flex ion source (Thermo Scientific), and column oven heater (Sonation). Samples were resuspended in 0.1% formic acid and directly loaded onto a 10 to 15 cm analytical capillary chromatography column with an integrated electrospray tip (~1 μm orifice), prepared and packed in house (50 μm ID × 20 cm & 1.9 μM C18 beads, ReproSil-Pur). Twenty-five percent of pMHC elution was injected for each analysis. Peptides were eluted using a gradient with 8 to 25% buffer B (70% Acetonitrile, 0.1% formic acid) for 50 min, 25 to 35% for 25 min, 35 to 55% for 5 min, 55 to 100% for 2 min, hold for 1 min, and 100 to 3% for 2 min.

Standard MS parameters were as follows: spray voltage, 2.0 kV; no sheath or auxiliary gas flow; heated capillary temperature, 275 °C. The Exploris was operated in DDA mode. Full-scan mass spectra (350–1200 m/z, 60,000 resolution) were detected in the orbitrap analyzer after accumulation of 3E^6^ ions (normalized AGC target of 300%) or 25 ms. For every full scan, MS^2^ were collected during a 3 s cycle time. Ions were isolated (0.4 m/z isolation width) for a maximum of 150 ms or 75% AGC target with the automatic maximum injection time setting enabled for parallelization. Ions were fragmented by HCD with 32% nCE at a resolution of 45,000. Charge states <2 and >4 were excluded, and precursors were excluded from selection for 30 s if fragmented n = 2 times within 20 s window.

### pTyr MS Data Acquisition

LC-MS/MS analysis of pTyr peptides was performed on an Agilent 1260 HPLC system coupled to an Orbitrap Exploris 480 mass spectrometer. Peptides were resuspended in 10 μl 0.1% acetic acid and loaded onto an analytical capillary column with an integrated electrospray tip (~1 μm orifice) prepared in house (50 μm ID × 12 cm with 5 μm C18 beads (YMC gel, ODS-AQ, 12 nm, S-5 μm, AQ12S05)). Peptides were eluted using a 140-min gradient with 13 to 42% buffer B (70% Acetonitrile, 0.2 M acetic acid) from 10 to 105 min and 42 to 60% buffer B from 105 to 115 min, 60 to 100% B from 115 to 122 min, and 100 to 0% B from 128 to 130 min at a flow rate of 0.2 ml/min with a flow split of approximately 10,000:1.

Standard MS parameters were as follows: spray voltage, 2.5 kV; no sheath or auxiliary gas flow; heated capillary temperature, 275 °C.

The mass spectrometer was operated in data-dependent acquisition with following settings for MS1 scans: m/z range: 350 to 2000; resolution: 60,000; AGC target: 3E^6^; auto IT: 50 ms. Within a 3 s cycle time, ions were isolated (0.4 m/z) and fragmented by HCD (nCE: 33%) with resolution: 60,000; AGC target: 1E^5^, max IT: 250 ms for all analyses except EGF-boost 500 ms (AGC target: 5E^5^, max IT: 500 ms). Unassigned and charge states <+2 and >+6 were excluded, and peptides were excluded from selection for 45 s if fragmented n = 2 times.

Crude peptide analysis was performed on a Q Exactive Plus hybrid quadrupole-orbitrap mass spectrometer coupled to an Agilent 1260 LC system to correct for variation in peptide loading across TMT channels using 2.5 kV no sheath or auxiliary gas flow; heated capillary temperature, 250 °C. Approximately 30 ng of the supernatant from pTyr IP was loaded onto an in-house packed precolumn (100 μm ID × 10 cm) packed with 10 μm C18 beads (YMC gel, ODS-A, AA12S11) connected in series to an analytical column (as previously described) and analyzed with a 75 min LC gradient [0–30% B from 0 to 40 min, 30–60% B from 40 to 50 min, 60–100% B from 50 to 55 min, and 100–0% B from 60 to 65 min]. MS1 scans were performed with m/z range: 350–2000; resolution: 70,000; AGC target: 3E^6^; max IT: 50 ms. The top ten abundant ions were isolated (isolation width 0.4 m/z) and fragmented (nCE = 33%) with 70,000 resolution, max IT 150 ms, AGC target 1E^5^. Unassigned, +1, and >+7 charge states were excluded, and dynamic exclusion was set to 30 s.

### MHC MS Search Space, Filtering, and Analysis

All mass spectra were analyzed with Proteome Discoverer (PD, version 2.5) and searched using Mascot (version 2.4) against the human SwissProt database (2021_01, 20,396 entries). No enzyme was used, precursor mass tolerance: 10 ppm, fragment mass tolerance: 20 mmu with TMT lot-specific isotopic correction factors applied. Variable modifications were set to include oxidized methionine, static modifications included N-terminal and lysine TMT.

Heavy leucine-containing peptides were searched for separately with heavy leucine (+7) as a dynamic modification against a custom database of the synthetic peptide standards. All analyses were filtered with the following criteria: search engine rank =1, isolation interference ≤30%, ion score ≥15 and percolator q-value ≤ 0.05. Master protein descriptions were used to assign source proteins to ambiguous peptides for downstream analyses. Reporter ion intensities of peptide spectrum matches (PSMs) assigned to the same peptide sequence were summed, and reporter ion intensities were corrected using hipMHC intensity values (CDK4/6i analysis only) as previously described (7). Only peptides with a length between 8 and 15 amino acids were considered for downstream analyses. Filtered PSMs and the hipMHC-corrected peptide-level datasets are included in supplemental Table S1.

To evaluate differences between conditions, the log_2_ transformed ratio of arithmetic mean intensity for drug- and DMSO-treated samples (n = 3) was calculated. To determine if peptides were significantly increasing/decreasing, an unpaired, two-sided *t* test was performed with *p* ≤ 0.05 set as the threshold for significance. PCA analyses were performed using MATLAB R2019b.

### pTyr MS Search Space, Filtering, and Analysis

All mass spectra were analyzed with PD 2.5 and searched using Mascot 2.4 against the human SwissProt database (version 2021_01, 20,396 entries). For pTyr analyses, spectra were searched using the following parameters: enzyme: trypsin, maximum missed cleavages: 2, precursor mass tolerance: 10 ppm, fragment mass tolerance: 20 mmu. Static modifications included TMT-10-labeled lysine and N-terminal residues, as well as cysteine carbamidomethylation. Dynamic modifications included methionine oxidation, and tyrosine, serine, and threonine phosphorylation.

Phosphorylation sites were localized with ptmRS module (17) with 216.04 added as a diagnostic mass for pTyr the immonium ion (18). Peptides were filtered with the following criteria: search engine rank =1, isolation interference ≤35%, ion score ≥17, and ≥1 tyrosine phosphorylated residue. Peptides were filtered with the following criteria: search engine rank = 1, isolation interference ≤35%, ion score ≥17, and ≥1 tyrosine phosphorylated residue. PSMs with >95% localization probability for all phosphorylation sites were classified as unambiguous and used for downstream analyses. We assessed underlabeling by searching data with variable TMT modifications and found <0.01% of filtered PSMs are unlabeled.

Crude peptide mixture was searched with the following parameters: enzyme: trypsin, maximum missed cleavages: 2, precursor mass tolerance: 10 ppm, fragment mass tolerance: 20 mmu. Static modifications included TMT-10-labeled lysine and N-terminal residues, as well as cysteine carbamidomethylation. Dynamic modifications included methionine oxidation. Peptides were filtered with the following criteria: search engine rank =1, ion score ≥20. Phosphotyrosine peptide reporter ion areas were corrected for variations in sample loading within each analysis using the median of peptide ratios in the crude peptide analysis for each channel relative to a selected reference channel. Next, reporter ion intensities were summed across matching PSMs. Filtered PSMs and the analyzed datasets are included in supplemental Table S2, where PSMs assigned as ambiguous/unambiguous are listed in separate tables. Hierarchical clustering and PCA analyses were performed using Matlab R2019b.

### Peptide MHC Binding Affinity

Binding affinity of pMHCs was estimated using NetMHCpan-4.0 against the allelic profile of SKMEL5 cells (19, 20). Only 9-mers were evaluated, and the minimum predicted affinity (nM) of each peptide was used to assign peptides to their best predicted allele. The threshold for binding was set at 500 nM.

### Enrichment Analyses

For pMHC pathway enrichment analyses, gene names from peptide source proteins were extracted and rank ordered according to the average log_2_ fold change over DMSO-treated cells. In cases where more than one peptide mapped to the same source protein, the maximum/minimum was chosen, depending on the directionality of enrichment analysis. We utilized gene set enrichment analysis (GSEA) 4.0.3 preranked tool against the Molecular Signatures Database hallmarks gene sets with 1000 permutations, weighted enrichment statistic (*p* = 1), and a minimum gene size of 15 for pMHC analyses (21, 22, 23). Results were filtered for FDR q-value ≤0.25, and nominal *p*-value ≤0.05.

### Experimental Design and Statistical Rationale

HipMHCs were titrated into six samples at three concentrations (n = 2) to generate a three-point calibration curve while minimizing protein input requirements. To compare two experimental conditions in the pMHC analyses (DMSO *versus* palbociclib treatment) and three experimental conditions (0s, 30s, 2m EGF stimulation) in pTyr analyses, n = 3 biological replicates were selected for each condition to allow for calculating statistical significance.

## Results

### Characterizing the Quantitative Accuracy of “Boosted” pMHC Analysis Using Synthetic, Heavy Isotope-labeled pMHCs

**Fig 1:**
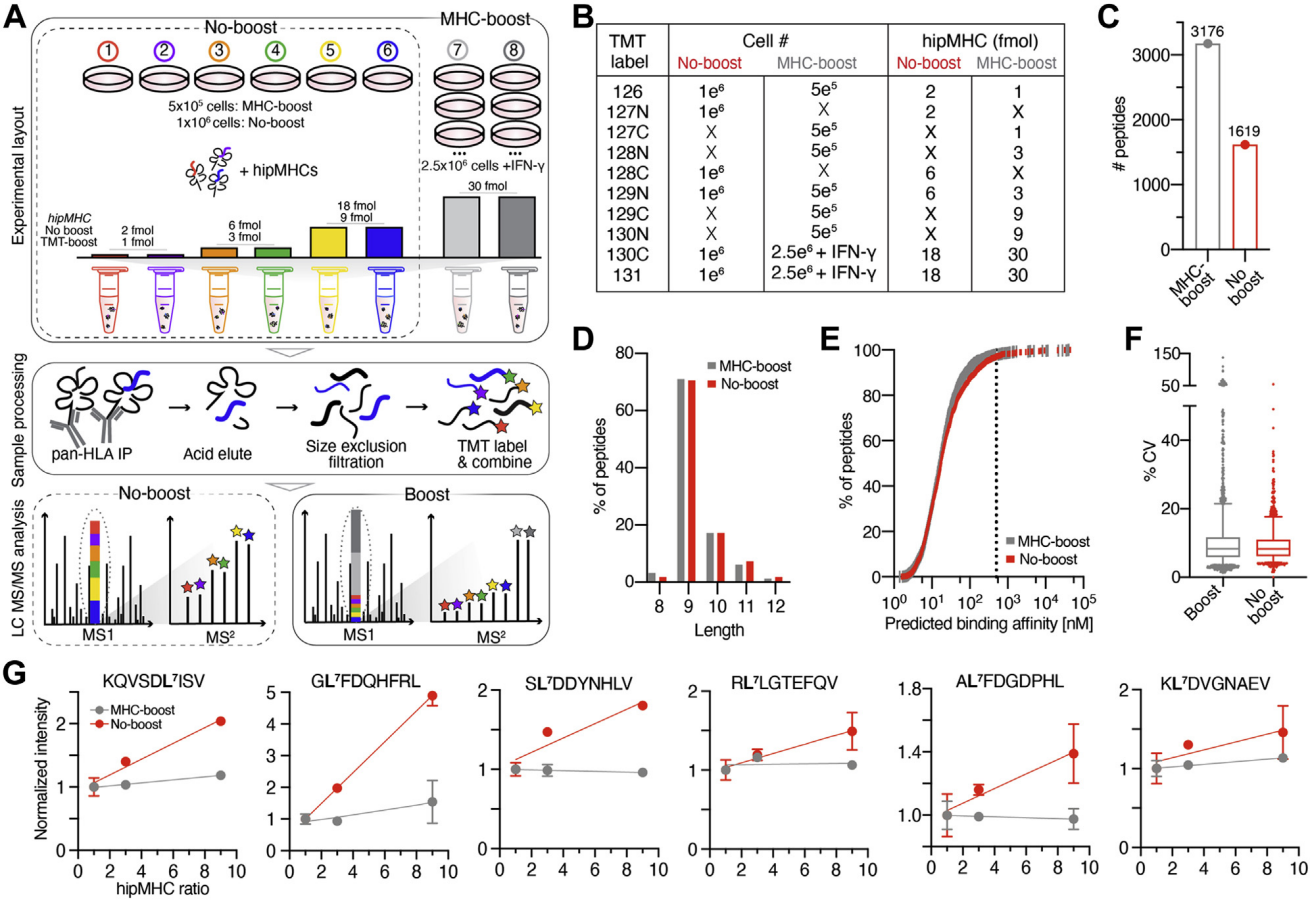
**Estimating ratio compression using hipMHCs in immunopeptidomic analyses.***A*, experimental setup of hipMHC quantitative immunopeptidomic analyses ± protein carrier. *B*, experimental setup of isobaric labels, cell number, and concentration of hipMHC added for each sample. *C*, number of unique pMHCs identified in a single analysis. *D*, length distribution of pMHCs. *E*, predicted binding affinity of 9-mers. 97.9% and 97.0% of 9-mers in the pMHC-boost and no-boost analyses, respectively, were predicted to have a binding affinity ≤500 nM (*dotted line*). *F*, coefficients of variation of pMHC-boost and no-boost analyses. Boxes outline the interquartile range, and whiskers the 5 and 95th percentiles. pMHC-boost median CV = 8.23%, no-boost = 8.30%. 95% PSMs have CV <17.7% (no-boost) and 21.5% (pMHC-boost), Violin plots of reporter ion intensities for pMHC-boost (*left*) and no-boost (*right*) analyses. Median: *black dashed line*, quartiles: *colored dotted line*. *G*, reporter ion intensities of hipMHC peptides normalized to the mean signal of the 1 fmol (pMHC-boost) or 2 fmol (no-boost) samples (y-axis), where the hipMHC ratio represents the amount of hipMHC added over the lowest concentration (x-axis). *Solid line* = linear fit, error bars show ± standard deviation. Source data can be found in supplemental Table S3.

To interrogate the impact of including a carrier proteome on pMHC identification and quantitation, we prepared a set of six cell-line-derived replicate samples comprised of 1 × 10^6^ cells per channel for the analysis without a protein carrier (“no-boost”), and a parallel experiment using 50% fewer cells per sample (5 × 10^5^ cells) for the “MHC-boost” analysis (Fig. 1, *A* and *B*). Empty channels were not incorporated to more closely mirror the experimental design of a previously reported study (10). As a protein carrier, we utilized two samples (two channels) of 2.5 × 10^6^ cells stimulated with 10 ng/ml interferon-gamma (IFN-γ) for 72 h. IFN-γ stimulation increases pMHCs levels approximately twofold, allowing for reduced cellular input requirements to generate approximately tenfold boost per sample. The inclusion of two carriers yields a combined signal-to-boost ratio of ~20-fold, in line with recent published guidelines for SCP experiments (supplemental Fig. S1, *A* and *B*) (12).

To measure ratio compression, we utilized a panel of six synthetic, heavy-isotope labeled pMHCs (hipMHCs), which were titrated into cell lysates prior to pMHC isolation to generate an internal standard curve against a consistent background immunopeptidome, as previously described (7). HipMHCs were added at a ratio of 1:1:3:3:9:9 across the six samples, with concentrations of 1, 3, and 9 fmol in the boost analysis, and proportionally, 2, 6, and 18 fmol in the no-boost analysis. The protein carrier samples contained 30 fmol of each hipMHC, tenfold more than the median concentration used across nonprotein carrier samples (Fig. 1*B*). After addition of hipMHCs, class I pMHC complexes were isolated from each sample by immunoprecipitation, acid elution, and size-exclusion filtration. Peptides for each sample were subsequently labeled with TMT, combined, and analyzed by LC-MS/MS.

As expected, including a protein carrier resulted in a large increase in the number of unique pMHC IDs using 50% less cellular input material for each channel: from a single injection using just 25% of the labeled mixture, 3176 unique pMHCs were identified in the MHC-boost sample, whereas 1619 were identified in the no-boost analysis (Fig. 1*C*). The peptides identified in both experiments followed expected length distributions (Fig. 1*D*), with 97.0% and 97.9% of 9-mers predicted to be allelic binders in no-boost and pMHC-boost analyses, respectively (Fig. 1*E*). While both analyses had equivalent median coefficients of variation (CV) across replicates (Fig. 1*F*), PSMs in the MHC-boost analysis had a wider distribution of CV values. Together, these data suggest that a 20× protein carrier improves the number of unique IDs while not altering peptide properties of the resultant data set but may result in slightly higher quantitative variation. We investigated whether the peptides with CVs ≥30% showed evidence of isotopic interference from the protein carriers in the TMT130N/TMT129C replicates but found that only a subset of peptides in TMT130N had increased reporter intensities compared with the TMT126. This trend was not reflected in the TMT129N channel and may be indicative of ion coalescence in the TMT130 reporter ions rather than isotopic interference (supplementary Fig. S1*C*) (12, 24). Of note, the proportion of missing values between the protein carrier and noncarrier samples in the pMHC boost analysis was comparable (4% of PSMs in no-boost, 8% in pMHC-boost), suggestive of sufficient ion sampling for a majority of peptides (supplemental Fig. S1*D*).

We next examined the intensity distributions across PSMs and found that the protein carrier samples had 3.5- to 4-fold higher intensity than the other samples in the boost analysis (supplemental Fig. S1*E*). Our expected intensity ratios were ~10:1 (fivefold increase in sample in the protein carrier channels, coupled to a twofold increase in MHC expression due to IFN-γ), thus the observed peptide ratios demonstrate a ~60% reduction in expected signal intensity, suggestive of ratio compression. Ratios of the titrated hipMHCs were subsequently analyzed, and substantial ratio compression was observed in both the MHC-boost and no-boost analyses (Fig. 1*G*). For example, in the no-boost analysis, the “GLFDQHFRL” peptide had a 1.8-fold reduction in dynamic range, while the “KLDVGNAEV” peptide had a 6.2-fold reduction, with the other hipMHC peptides falling between these two extremes. While the hipMHC intensity ratios did not match expected values in the no-boost analysis, reporter ion intensities did increase with increasing concentration of hipMHC. By comparison, the quantitative accuracy in the MHC-boost analysis was severely negatively affected by the presence of the protein carriers, as there was minimal difference in the reporter ion intensities for the hipMHC standards across all samples, with “GLFDQHFRL,” being the only exception (6.7-fold reduction in observed *versus* expected dynamic range). Taken together, these data demonstrate that while ratio compression exists in non-boost and boost experiments alike, likely due to the dense background of the endogenous immunopeptidome, the presence of a protein carrier exacerbated this effect to the extent that pMHCs up to ninefold higher in concentration could not be differentiated *via* isobaric intensities. It is worth noting that hipMHCs were added at relatively high concentrations, representing a range of ~1000 to 10,000 pMHCs/cell. Quantitative accuracy of endogenous pMHCs at lower presentation levels may be further negatively impacted by the presence of a protein carrier.

### Protein Carrier Channel Skews Biological Interpretation of Palbociclib-induced pMHC Repertoire Alterations

**Fig 2:**
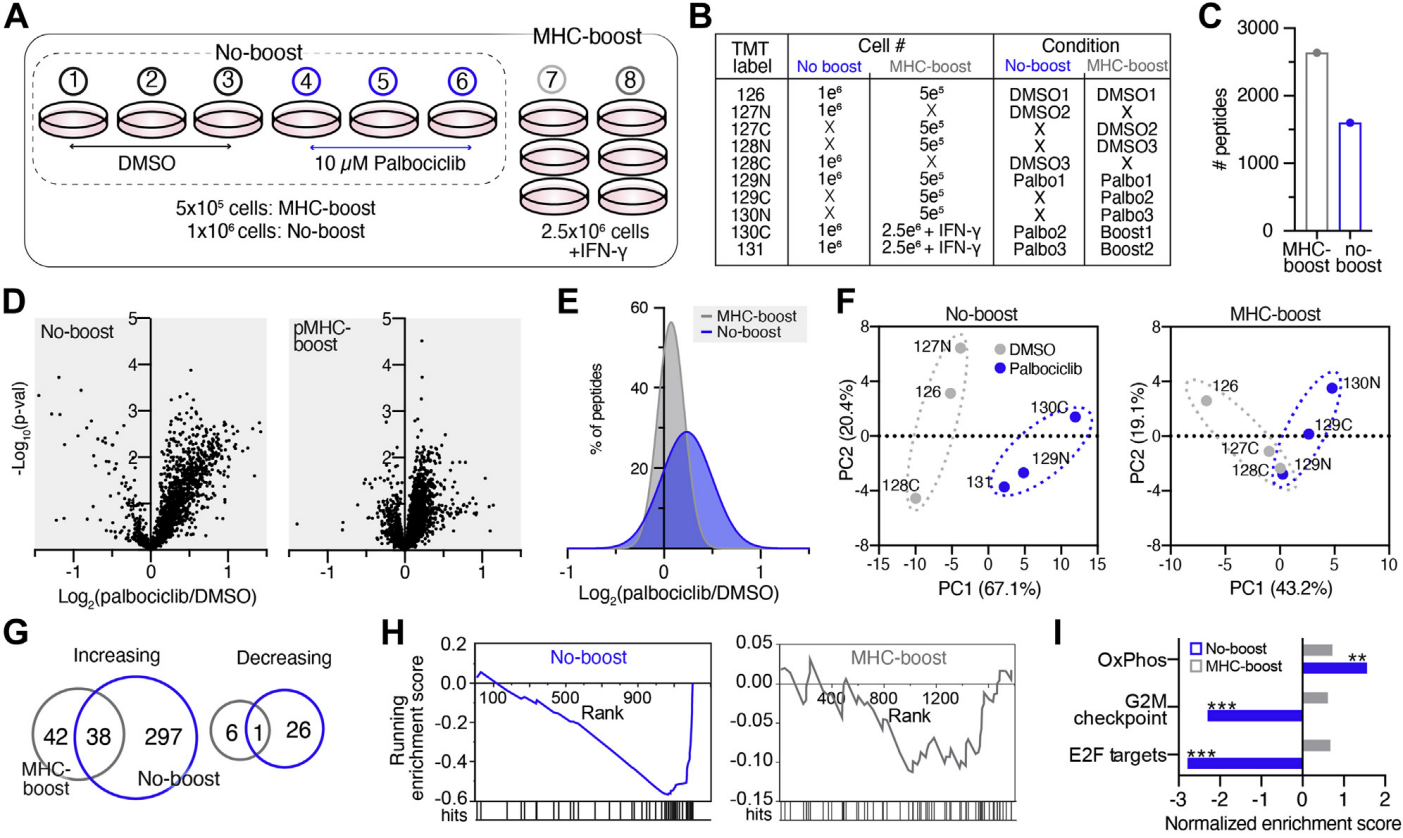
**Palbociclib-induced pMHC repertoire alterations are masked in the presence of a protein carrier.***A*, experimental setup of pMHC analyses ± protein carrier with 72 h DMSO or 10 μM palbociclib treatment. *B*, experimental setup of isobaric labels, cell number, and treatment conditions. *C*, number of unique peptides identified in pMHC-boost (2637) and no-boost (1602) analyses. *D*, volcano plot displaying the log_2_(palbociclib/DMSO) of pMHCs (x-axis), where the fold change is calculated from the mean intensity of *n* = 3 biological replicates per condition, *versus* significance (y-axis, mean adjusted *p*-value, unpaired two-sided *t* test). *E*, histogram distribution of unique pMHC fold change in expression. *F*, samples plotted by principal component 1 (PC1) and PC2 score for no-boost (*left*) and pMHC-boost (*right*) analysis, colored by treatment condition. Percentages are % variance explained by the plotted PC. *G*, Venn diagram of peptides significantly increasing (*upper*) and decreasing (*lower*) with palbociclib treatment in the no-boost (*blue*) and pMHC-boost (*gray*) analyses. *H*, pMHC enrichment plots for E2F targets for the no-boost (*gray*, *p* = 0.13, q = 0.88) and pMHC-boost (*blue*, *p* < 0.001, q < 0.001) analyses. Hits mark pMHCs of source proteins mapping to E2F targets. *I*, normalized enrichment scores from enrichment analyses of pMHC-boost (*gray*) and no-boost (*blue*) datasets. Positive/negative scores represent directionality of pathway enrichment. Significant enrichment is noted by ∗∗*p* < 0.01, ∗∗∗*p* < 0.001, with FDR-q values <0.25.

To further assess the accuracy of quantifying endogenous pMHCs in the presence of a protein carrier in a biological context, we evaluated whether a carrier proteome would affect data interpretation of melanoma cells treated with the CDK4/6 inhibitor, palbociclib, which increases pMHC presentation and induces palbociclib-specific repertoire changes, as previously reported (7). Cells were treated with 10 μM palbociclib or DMSO as a vehicle control for 72 h in triplicate and analyzed alone or with an IFN-γ stimulated protein carrier channel for a combined 20-fold signal-to-boost ratio, using a similar setup to the previous experiment. (Fig. 2, *A* and *B*).

Similar to the hipMHC experiment, “boosting” with a protein carrier yielded a greater number of unique peptides identified (2637 in the “MHC-boost” analysis *versus* 1602 in the “no-boost” analysis) (Fig. 2*C*), with similar length distributions (supplemental Fig. S2*A*). Of note, while IFN-γ stimulation has been shown to augment the presentation of IFN-γ-related peptides, the peptides identified only in the MHC-boost analysis did not show an enrichment for IFN-γ-related source proteins, suggesting this boosting strategy did not substantially skew the detected repertoire outside of typical run-to-run variation.

The no-boost experiment recapitulated our previously reported results (7), where a majority of peptides showed an slight increase in presentation levels following palbociclib treatment (median fold change 1.17×), while peptides in the MHC-boost experiment showed a narrower distribution of changes, centered around a median fold change of just 1.05× (Fig. 2, *D* and *E*). In line with this finding, PCA showed superior separation of DMSO and palbociclib-treated samples in the no-boost *versus* the MHC-boost analysis (Fig. 2*F*).

To interrogate the data further, we considered the 1092 unique peptides quantified in both analyses (supplemental Fig. S2*B*). Of these peptides, fewer peptides were significantly increasing or decreasing in presentation in the MHC-boost analysis compared with the non-boost analysis (Fig. 2*G*), masking biological interpretation of the data. For example, 334 common peptides significantly increased in presentation in the non-boost analysis, while only 80 common peptides in the boost analysis significantly increased.

Interestingly, 42 of the 80 peptides were significantly increased in only the MHC-boost but not the no-boost analysis. Upon closer inspection, we found 76% of peptides also showed in increase in presentation in the no-boost analysis but did not achieve statistical significance. Ratio compression can reduce variation in reporter-ion intensities, which we observed as reduced median coefficients of variation in the MHC-boost analysis compared with the no-boost analysis (supplemental Fig. S2*C*). This may artificially increase the likelihood of statistical significance among replicate samples, offering a possible explanation for this finding.

We next evaluated whether the altered quantitation in the boost analysis would change the previously described key findings of this experiment, namely that MHC peptides derived from proteins in pathways known to be perturbed by CDK4/6 inhibition show significant positive enrichment (oxidative phosphorylation, OxPhos) and negative enrichment (G2M checkpoints and E2F targets) (7). To this end, we performed an enrichment analysis using the MSigDB Hallmarks gene set database by rank ordering the gene names for pMHC source proteins in decreasing order of fold-change (21, 22, 23). In the no-boost analysis, 10 μM palbociclib treatment showed significant enrichment in OxPhos, G2M checkpoints, and E2F targets, mirroring previously reported findings (Fig. 2, *H* and *I*). In contrast, no pathways, including the three highlighted in the no-boost analysis, showed significant enrichment using the MHC-boost dataset data. A comparison of E2F target peptides between the analyses illustrates this finding—most peptides with decreased expression in the no-boost analysis showed little change in expression in the presence of a protein carrier. (supplemental Fig. S2*D*). These data reaffirm that while utilizing a protein carrier channel can increase the number of peptides identified and quantified across samples using lower cellular input, enhanced ratio compression due to the presence of a protein carrier can alter quantitative accuracy to the extent that known biological findings are masked, hiding relevant insight.

### Effects of PV-stimulated Protein Carrier on Quantitative Phosphotyrosine Analyses

**Fig 3:**
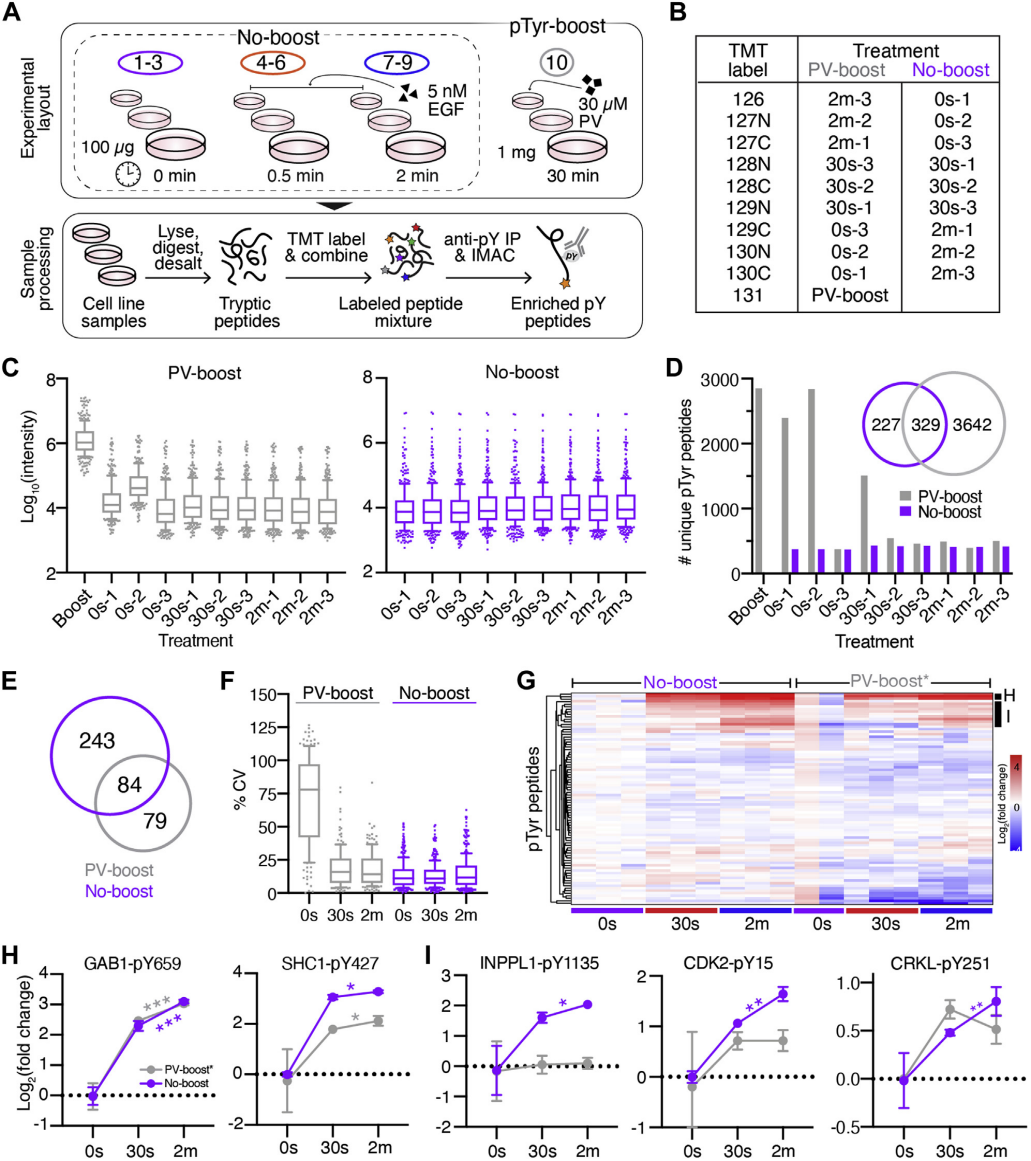
**Analysis of EGF-induced signaling dynamics in the presence of a PV-treated protein carrier.***A*, schematic of PV-boost *versus* no-boost experimental layout and pTyr peptide enrichment. *B*, isobaric labeling scheme and treatment conditions. *C*, reporter ion intensities for PV-boost (*left*) and no-boost (*right*) analyses. *Boxes* outline the interquartile range, and whiskers the 10 and 90th percentiles. *D*, number of unique pTyr peptides quantified in each sample. Venn diagram shows overlap in total pTyr peptides between analyses. *E*, Venn diagram of the number of unique pTyr peptides quantified across all samples in each analysis (no missing values). *F*, coefficients of variation of PV-boost (*gray*) and no-boost (*purple*) analyses. *Boxes* outline the interquartile range, and whiskers the 10 and 90th percentiles. PV-boost median CV: 14 to 78%, no-boost: 11 to 12%. *G*, hierarchical clustering (Euclidean) of Log_2_(fold change) values of pTyr sites identified in both analyses. PV-boost∗ values represent the original values from the PV-boost analysis renormalized to the mean 0s intensity with the 0s-2 excluded. Source data can be found in supplemental Table S4. *H* and *I*, Log_2_(fold change) values of peptides in clusters *H* and *I* (Fig. 3*G*). Significance values: two-tailed *t* test of 30s *versus* 2m time point. ∗*p* < 0.05, ∗∗*p* < 0.01, ∗∗∗*p* < 0.001. Error bars represent ± standard deviation.

Since the effect of boosting appeared to adversely affect quantitative accuracy in the immunopeptidomics experiments, we sought to evaluate whether utilizing a protein carrier would also impact quantitative accuracy in pTyr analyses. To provide a set of samples with altered signaling of a biologically relevant network for quantification, we utilized A549 cells stimulated with 5 nM EGF for 0 s, 30 s, or 2 min (0s, 30s, 2m) to drive a dynamic response in tyrosine phosphorylation levels among a subset of epidermal growth factor receptor (EGFR)-related pTyr sites, as previously described (14, 16, 25). Three biological replicates of 100 μg input material for each time point were utilized in the “no-boost” analysis, whereas the “PV-boost” analysis contained the same replicate samples along with 1 mg of protein carrier, A549 cells stimulated with PV to halt tyrosine phosphatase activity, thereby driving elevated pTyr signal (Fig. 3*A*). Peptide amounts and the labeling scheme were selected to match the upper and lower limits of sample input utilized by a previously reported pTyr boosting study (Chua *et al.*); however, we utilized a lower concentration of pervanadate (30 μM *versus* 500 μM) (5). Following tryptic digestion and standard sample processing, samples were labeled with TMT-10plex, and tyrosine-phosphorylated peptides were subsequently purified using two-step enrichment followed by LC-MS/MS analysis (Fig. 3, *A* and *B*) (16, 25).

Even though we treated cells with a lower concentration of PV relative to Chua *et al.*, the PV-treated protein carrier sample still had substantially higher reporter ion intensities (~100-fold) compared with the EGF-stimulated samples, well outside the suggested protein carrier-to-signal range recommended for SCP boost experiments (Fig. 3*C*). Indeed, the high signal level from the TMT-131 labeled PV-boost protein carrier channel resulted in isotopic interference in the second replicate of the zero-second channel (0s-2) labeled with the 130N TMT tag (supplemental Fig. S3*A*). By comparison, the no-boost analysis showed similar intensity distributions across samples.

As anticipated, the PV-boost analysis identified a considerably higher number of unique pTyr peptides compared with the no-boost analysis (3971 *versus* 556) (Fig. 3*D*). However, a majority of identified peptides in the PV-boost analysis were only quantified in the protein carrier channel or adjacent channels (isotopic interference), resulting in a large number of PSMs with missing values (up to 94%). By comparison, the no-boost analysis had far fewer peptides with missing values (up to 17%). Consequently, despite the greater number of overall pTyr-peptide identifications, the PV-boost analysis contained just 163 pTyr peptides quantifiable across all samples *versus* 327 in the no-boost analysis, reducing overall data quantity by twofold (Fig. 3*E*). The number of EGFR signaling related peptides was similarly reduced with 40 *versus* 20 pTyr peptides mapping to proteins in KEGG ErbB signaling pathway in the no-boost and PV-boost analyses, respectively (26).

To assess whether the PV-treated protein carrier channel also influenced the accuracy of the quantitative temporal signaling data, we compared the coefficients of variation between analyses (Fig. 3*F*). The 30s and 2m time points showed slightly higher variability in the PV-boost analysis *versus* the no-boost analysis, where, for example, the median 30s CV was 11% in the no-boost analysis compared with 16% in the PV-boost analysis. The 0s time point in the PV-boost analysis exhibited high CVs as a result of the isotopic interference (median 78%), greatly altering quantitative accuracy.

To compare the quantitative dynamics between analysis, a hierarchical clustering analysis of the 84 peptides quantified in both analyses was performed (supplemental Fig. S3*A*). The 0s-2 sample with isotopic interference greatly skewed quantitation by increasing the mean 0s signal, thus most of the peptides in the PV-boost analysis appear to have decreased phosphorylation in response to EGF, as compared with the no-boost analysis where the same sites show constant phosphorylation levels (supplemental Fig. S3*B*). Moreover, the increase in the mean 0s quantitation in the PV-boost analysis resulted in substantial ratio compression among peptides modulated by EGF stimulation (supplemental Fig. S3, *C* and *D*).

To better assess the effects of the PV-boost protein carrier on quantitative accuracy, we removed the 0s-2 data labeled with 130N, after which the quantitative dynamics more closely mirrored those of the no-boost condition (Fig. 3*G*). Of note, the 0s-1 (130C) sample still showed higher pTyr levels than the 0s-3 (129C) sample, suggestive of ion coalescence from 0s-2 (130N).

Several of the EGF-modulated peptides showed a large increase in phosphorylation after stimulation, and while a few peptides showed correlated dynamics such as GAB1-pY659, which also had one of the largest dynamic changes in tyrosine phosphorylation, others still showed dynamic range suppression in the PV-boost analysis (Fig. 3*H*). For example, we measured an 11-fold increase in pTyr for SHC1-pY427 following 2 min of EGF stimulation in the no-boost analysis, which was reduced to a fourfold change when analyzed with a protein carrier. While the same trend of increased phosphorylation with EGF stimulation was preserved between the analyses for the SHC1 peptide, subtler pTyr changes may be masked by the effects of ratio compression from precursor interference. This was seen in the INPPL1-pY1135, CDK2-pY15, and CRKL-pY251 peptides, which have significantly different pTyr levels between the 30s and 2m time points in the no-boost analysis but are not significantly different in the PV-boost dataset (Fig. 3*I*). Indeed, 33 peptides have significantly different pTyr levels (*p* ≤ 0.05) between the 30s and 2m condition in the no-boost analysis *versus* just 20 in the PV-boost analysis (supplemental Fig. S4*A*), indicative of increased dynamic range suppression. PCA reinforces this finding, as the 30s and 2m samples cluster closer together (regardless of inclusion/exclusion of the 0s-2 sample), whereas the no-boost samples cluster with superior separation (supplemental Fig. S4*B*). While normalizing data to phosphopeptides that remain unchanged in response to EGF stimulation offers a minor improvement in PV-boost quantitative accuracy (supplemental Fig. S4*C*), this strategy requires a-priori knowledge of “housekeeping” phosphosites and is therefore not an applicable strategy for analyzing samples with an unknown pTyr dynamics.

### Reduction in Protein Carrier Improves Quantitative Accuracy but Still Increases Missing Values

**Fig 4:**
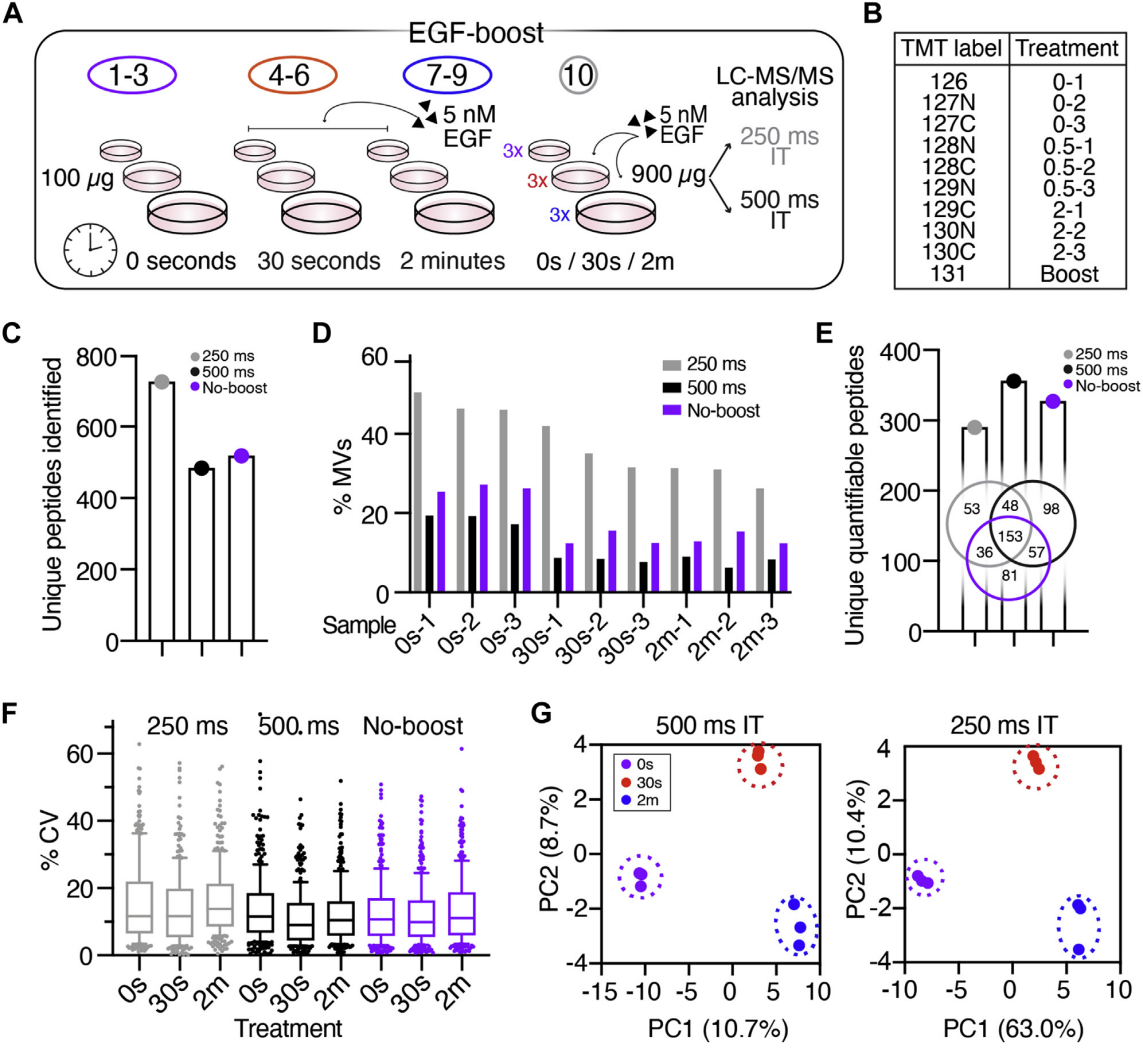
**pTyr-boost with 9× EGF-boost protein carrier improves quantitative accuracy but still yields large number of missing values.***A*, experimental layout of EGF-boost experiment with 9× protein carrier. *B*, isobaric labeling scheme and treatment conditions. *C*, total number of unique pTyr peptides identified in each analysis. *D*, proportion of PSMs with missing values for each sample. *E*, total number of unique pTyr peptides quantified in each analysis and Venn diagram of peptides commonly identified between analyses (no MV’s). *F*, coefficients of variation between replicates in EGF-boost and no-boost analyses. *Boxes* outline the interquartile range, and whiskers the 10 and 90th percentiles. EGF-boost 250 ms median CV: 12 to 14%, 500 ms IT: 9 to 12%, no-boost: 10 to 11%. *G*, samples plotted by principal component 1 (PC1) and PC2 score, colored by EGF stimulation condition for 500 ms EGF boost and no-boost analyses. Percentages describe the variance explained by the plotted PC.

To determine whether we could improve quantitative accuracy and overall data quality by using a smaller amount of a more targeted boost channel, we performed two additional experiments using 100 μg in triplicate of the 5 nM EGF stimulated samples at 0, 30s, and 2m time points used in the PV-boost/no-boost experiments along with a 900 μg protein carrier consisting of equal parts of each sample for a boost-to-signal ratio of approximately ninefold (“EGF-boost”) (Fig. 4, *A* and *B*). Unlike the PV-boost, which inhibits tyrosine phosphatases and results in a general, though variable, increase in most phosphorylated tyrosines (supplemental Fig. S5*A*), we hypothesized that using EGF-stimulated samples as a boost would lead to more targeted detection of the EGFR signaling network. Additionally, to assess whether increased ion numbers might yield improved quantitative accuracy and fewer missing values, the EGF-boost analyses were analyzed under two conditions: at an AGC target of 1E^5^ and maximum IT of 250 ms, as performed in the PV-boost/no-boost analyses, and with an increased AGC target of 5E^5^ and maximum IT of 500 ms.

As expected, an increased number of unique pTyr peptides were identified in the 250 ms analysis compared with the 500 ms analysis (Fig. 4*C*, supplemental Fig. S5*B*). However, the proportion of PSMs with MVs was similarly increased in the 250 ms analysis (250 ms: 26–50% MVs, 500 ms: 6–19% MVs), resulting in fewer unique pTyr peptides quantifiable across all samples in the 250 ms analysis (290) *versus* the 500 ms analysis (356) (Fig. 4, *D* and *E*). In comparison to the 327 pTyr peptides identified and quantified in the no-boost analysis previously described, the 500 ms IT “EGF-boost” offers a slight increase in data quantity. In both EGF-boost analyses, we identified 45 EGFR-related peptides quantified across all samples, representing a slight improvement over the no-boost data (40) and more than double the EGF-related peptides identified in the PV-boost data. This finding is in support of our hypothesis that an EGF-stimulated protein carrier would enhance data quantity of EGF signaling-related peptides.

We next compared intensity distributions for each of the nine EGF-stimulated samples to evaluate data quality and found them to be similar in both EGF-boost analyses with no obvious isotopic interference from the protein carrier, which had an increased intensity distribution near the expected ninefold ratio (supplemental Fig. S5*C*). We verified this by comparing the CVs between replicates and found that the 500 ms IT and no-boost analyses had comparable median CVs (10.4% and 10.6%, respectively), whereas the 250 ms IT analysis had a lightly higher median CV (12.4%) (Fig. 4*F*). Nevertheless, a PCA analysis showed clear separation of samples by treatment condition in both EGF-boost analyses, a substantial improvement over the PV-boost analysis (Fig. 4*G*).

**Fig 5:**
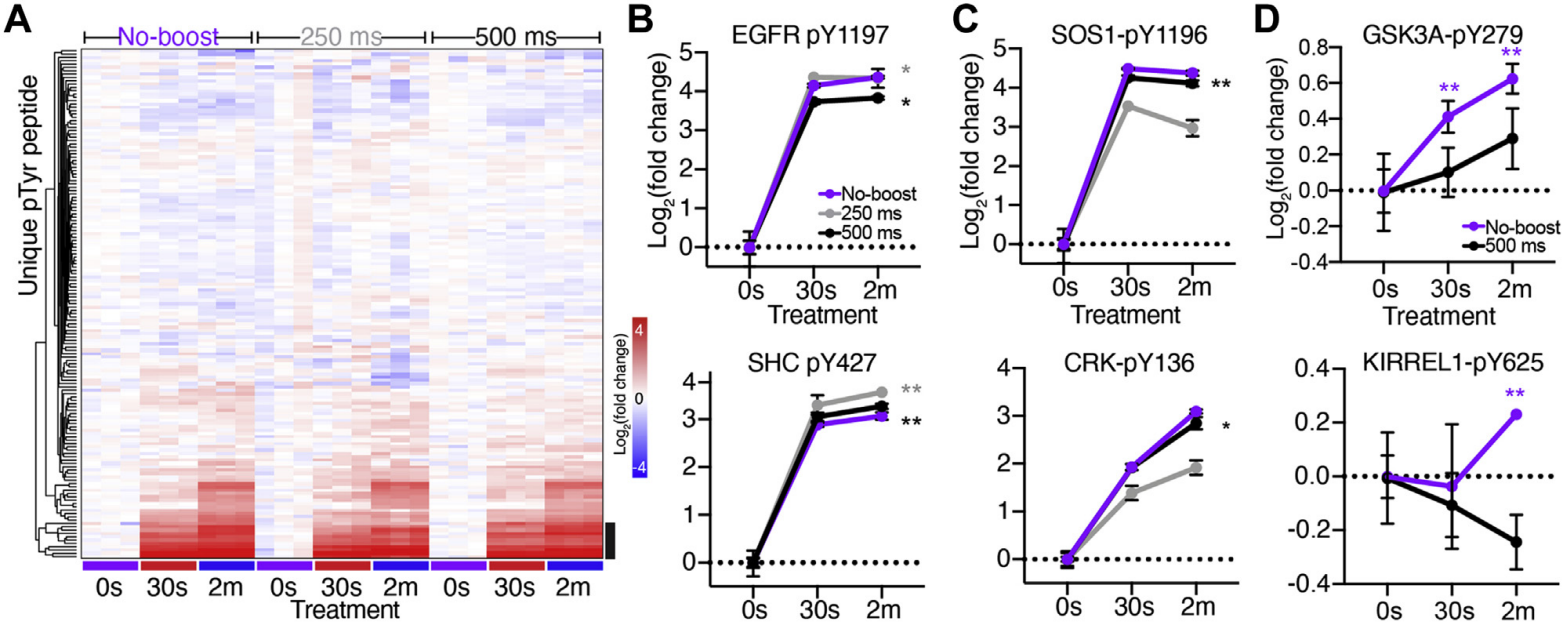
**9× EGF-stimulated protein carrier has higher quantitative accuracy, but EGF-modulated sites still show altered pTyr dynamics and ratio compression.***A*, hierarchical clustering of peptides identified in all three analyses, represented as log_2_(fold change) of each sample normalized to the mean reporter ion intensity of the 0s control per analysis. *Black bar* highlights EGF-modulated peptides highlighted in *B* and *C*, supplemental Fig. S5*C*. Source data can be found in supplemental Table S5. *B* and *C*, Log_2_(fold change) of pTyr signal of selected peptides from clusters *B* and *C* highlighted in *A*. Error bars represent ± standard deviation (n = 3). Pearson correlation significance (two-tailed): ∗*p* < 0.05, ∗∗*p* < 0.01. *D*, Log_2_(fold change) pTyr signal of peptides. Significance values: two-tailed *t* test of 0s *versus* 30s/2m time point. ∗∗*p* < 0.01, Error bars represent ± standard deviation.

To further assess quantitative accuracy, the 153 peptides commonly identified and quantified across the three analyses were hierarchically clustered (Fig. 5*A*), displaying similar patterns of phosphorylation with EGF-responsive peptides clustering together (Fig. 5*A* and supplemental Fig. S5*D*). While some of the peptide had significantly correlated phosphorylation dynamics between the no-boost and EGF-boosted analyses (Fig. 5*B*), others showed significant correlation only in the no-boost and 500 ms IT condition (Fig. 5*C*). Despite this finding, even in the 500 ms dataset, fewer peptides showed a significant change in phosphorylation from the 0s control at the 30s and 2m time points compared with the no-boost analysis (supplemental Fig. S5*E*). Of the peptides that were not significant in the 500 ms analysis, there was evidence of ratio compression and altered quantitative dynamics in comparison to the no-boost analysis (Fig. 5*D*), highlighting that not all peptides had comparable quantitation between the two experiments.

Together, these data demonstrate that a smaller and more targeted carrier-to-signal ratio may improve quantitative accuracy compared with a larger protein carrier, especially when coupled with longer ion accumulation times for better ion statistics on the nonboosted channels. However, the smaller protein carrier offers only a slight benefit in data quantity and still demonstrates reduced quantitative accuracy compared with the no-boost control, even when using instrument parameters designed to improve accuracy.

## Discussion

The ability to redce sample input and/or increase signal with a protein carrier is particularly appealing in immunopeptidomics and tyrosine phosphoproteomics, two applications that are often limited by larger sample requirements. However, our data indicate that inclusion of a protein carrier decreases quantitative accuracy in MS^2^-based quantitative analyses, even when using a signal-to-boost ratio within SCP guidelines (20×) (12). Loss of quantitative accuracy associated with “boosting” manifested as high ratio compression in pMHC analyses that masked dynamic alterations in pMHC expression levels and obscured known biological findings. Ratio compression was similarly observed in pTyr analyses, with the degree of ratio compression amplified with increasing signal in the protein carrier channel. Ratio compression in pMHC analyses may be attributed to the high background of peptides coeluting with similar sequences, as even the no-boost analysis and label-free analyses show evidence of ratio compression, though to a lesser degree (7).

To offset ratio compression and thus improve quantitative accuracy in “boosted” sample analyses, triple-stage mass spectrometry (MS^3^) and high-field asymmetric waveform ion mobility spectrometry (FAIMS) have been shown to reduce ratio distortion, although both methods can come at a cost of sensitivity and data quantity (27, 28). Additional experiments, similar in format to those described here, will be useful in determining whether MS^3^ and/or FAIMS can offer improved quantitative accuracy without compromising data quantity in this setting. To enable such comparisons, hipMHCs provide a useful tool to evaluate ratio compression in place of exogenously added peptide standards.

Ratio compression was similarly observed in pTyr analyses, with the degree of ratio compression amplified with increasing signal in the protein carrier channel. It is worth noting that while MS^3^ may be applicable to improve quantitative accuracy for pMHC analysis, it is a relatively unattractive solution for tyrosine phosphoproteomics due to the cost in sensitivity, lower precision, and fewer peptide identifications compared to MS^2^ (29).

In pTyr analyses, utilizing a PV-treated protein carrier provided a strong increase in MS1 signal and greatly increased the number of pTyr peptides identified. Unfortunately, large proportions of missing values in this analysis decreased the overall data quantity compared with a parallel analysis performed without the protein carrier, likely due to under sampling of the ion populations of noncarrier samples. While Chua *et al.* were able to replace missing values by interpolation, this strategy is not applicable for analyzing biological systems where the quantitative dynamics are unknown. In addition to missing values, the high signal level of the PV-boost protein carrier resulted in isotopic interference in adjacent channel(s) that negatively impacted quantification of these channels and their respective conditions. These channels could be removed in postprocessing to improve quantitative accuracy or excluded altogether, though this approach decreases the number of TMT tags available for sample multiplexing, diminishing the throughput and utility of this approach. Furthermore, dynamic range suppression was still observed even after excluding the sample with highest isotopic interference, suggesting that a boost-to-signal ratio of this magnitude may adversely affect the quantitative accuracy of the experiment regardless of isotopic leakage.

Decreasing the magnitude of the protein carrier in pTyr analyses and increasing the maximum IT and AGC target for increased ion sampling of the noncarrier samples decreased MVs compared with the PV-boost analysis and increased the total number of identified and fully quantified peptides by 29 compared with the no-boost analysis. Despite this slight improvement in quantifiable phosphopeptides, some peptides still showed altered dynamics and ratio compression relative to the no-boost analysis, suggesting that use of a protein carrier in this experimental design is of little benefit. Further increasing the AGC target/IT may improve quantitative accuracy but will likely reduce the number of scans acquired and thus the number of identified peptides, as nearly all PSMs reached the maximum injection time in the PV-boost and EGF-boost analyses (supplemental Fig. S6). Optimizing acquisition parameters to more closely sample the chromatographic elution near the apex (30) may offer some improvement in missing values and quantitative accuracy by increasing ion abundance of noncarrier ions within selected fill times, though the balance between data quantity and data quality across acquisition settings remains to be thoroughly explored. Alternatively, decreasing the number of multiplexed samples would increase ion sampling of noncarrier samples, but limited multiplexing would further reduce the utility of the assay.

These data illustrate that experiments leveraging protein carriers should rigorously evaluate the quantitative impact of the protein carrier (namely ion suppression, ion coalescence, ratio compression, missing values, coefficients of variation, and isotope leakage) to avoid misinterpretation of biological data. As several studies have documented (11, 31), and as supported by our findings here, selecting a protein carrier that is similar to one or more of the noncarrier samples may improve targeting of relevant peptides while also improving quantitative accuracy and offering the potential for improved normalization. At the same time, application of protein carriers to clinical proteomics or single-cell proteomics necessitates utilization of a carrier that is distinct from any of the noncarrier channels. In these cases, our data suggest caution in interpreting the quantification in these analyses. Future studies exploring alternative instrument acquisition parameters and configurations, along with protein carrier magnitudes and signal stimulation strategies, will further illuminate whether protein carriers can be effectively used for quantitative studies in these applications, or whether improvements in sample preparation and instrument sensitivity may pave an alternative path forward in achieving high-accuracy, high-precision measurements without a signal boost.

## Data Availability

The MS proteomics data have been deposited to the ProteomeXchange Consortium *via* the PRIDE partner repository with the dataset identifier PXD025073. This article contains supplemental data, including the data matrixes, search results, and associated PRIDE filenames used for the results described in this study.

## Supplemental data

This article contains supplemental data.

## Conflict of interest

The authors declare no potential conflicts of interest.
