# Supplementary material for: Quantitative Consequences of Protein Carriers in Immunopeptidomics and Tyrosine Phosphorylation MS2 Analyses
Source: Mol Cell Proteomics. 2021 May 28;20:100104. doi: 10.1016/j.mcpro.2021.100104 (PMC8240026; doi:10.1016/j.mcpro.2021.100104)
Supplement: Supplemental Figures S1–S6 [file mmc1.pdf]

Quantitative consequences of protein carriers in immunopeptidomics and tyrosine phosphorylation MS^2 analyses

Lauren E. Stopfer, Jason E.Conage-Pough & Forest M. White.

Supplemental Data

Supplemental Figures 1-6

Supplementary Tables 1-5 captions

SUPPLEMENTAL FIGURES

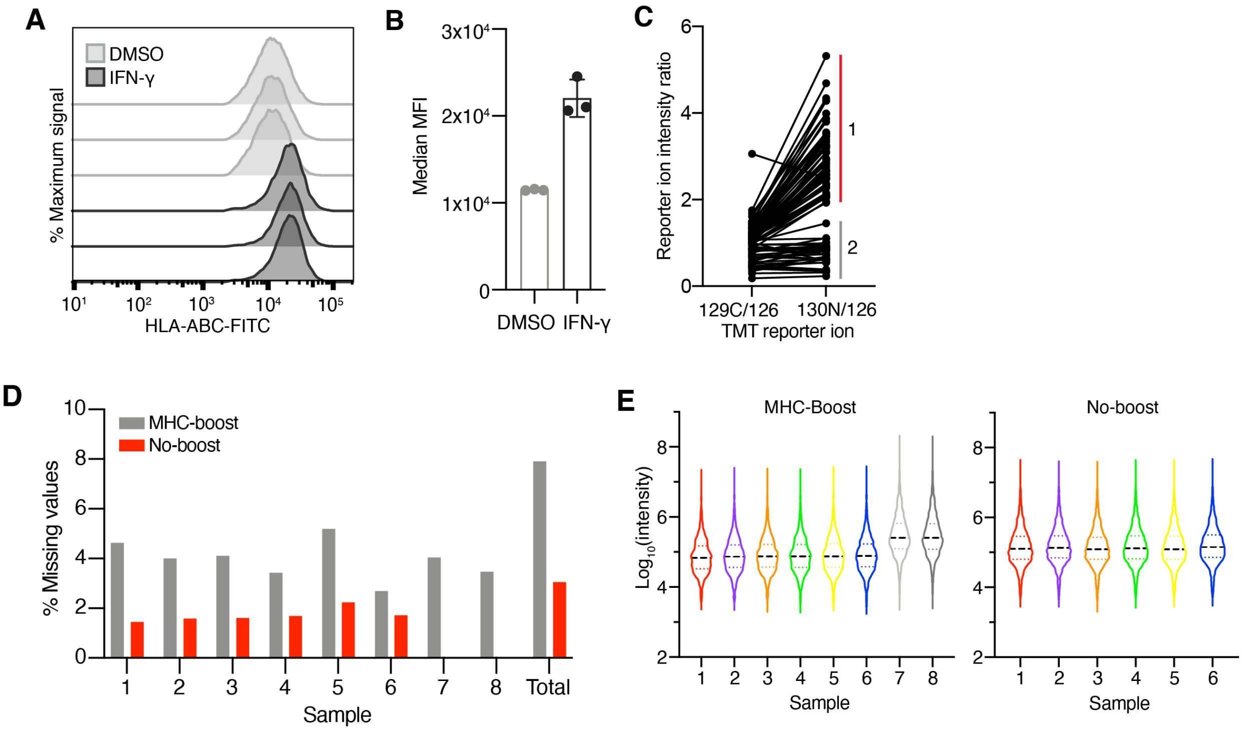

Supplemental Fig. S1.

A, Flow cytometry analysis of surface HLA expression in SKMEL5 cells after 72h of DMSO or IFN-g treatment. Data are presented as % of maximum fluorescence intensity signal. N=3 biological replicates per treatment condition.

B, Median MFI of flow cytometry measurements in B. Error bars represent standard deviation from n=3 biological replicates.

C, TMT reporter ion intensity ratios between TMT129C and TMT130N versus TMT126 in MHC-boost for peptides with >30% coefficients of variation. Peptides in cluster 1 (red) show altered quantitation in TMT130N only, whereas peptides in cluster 2 (grey) have comparable quantitation.

D, Percentage of total PSMs with missing values in each sample.

E, Violin plots of reporter ion intensities for pMHC-boost (left) and no-boost (right) analyses. Median: black dashed line, quartiles: colored dotted line.

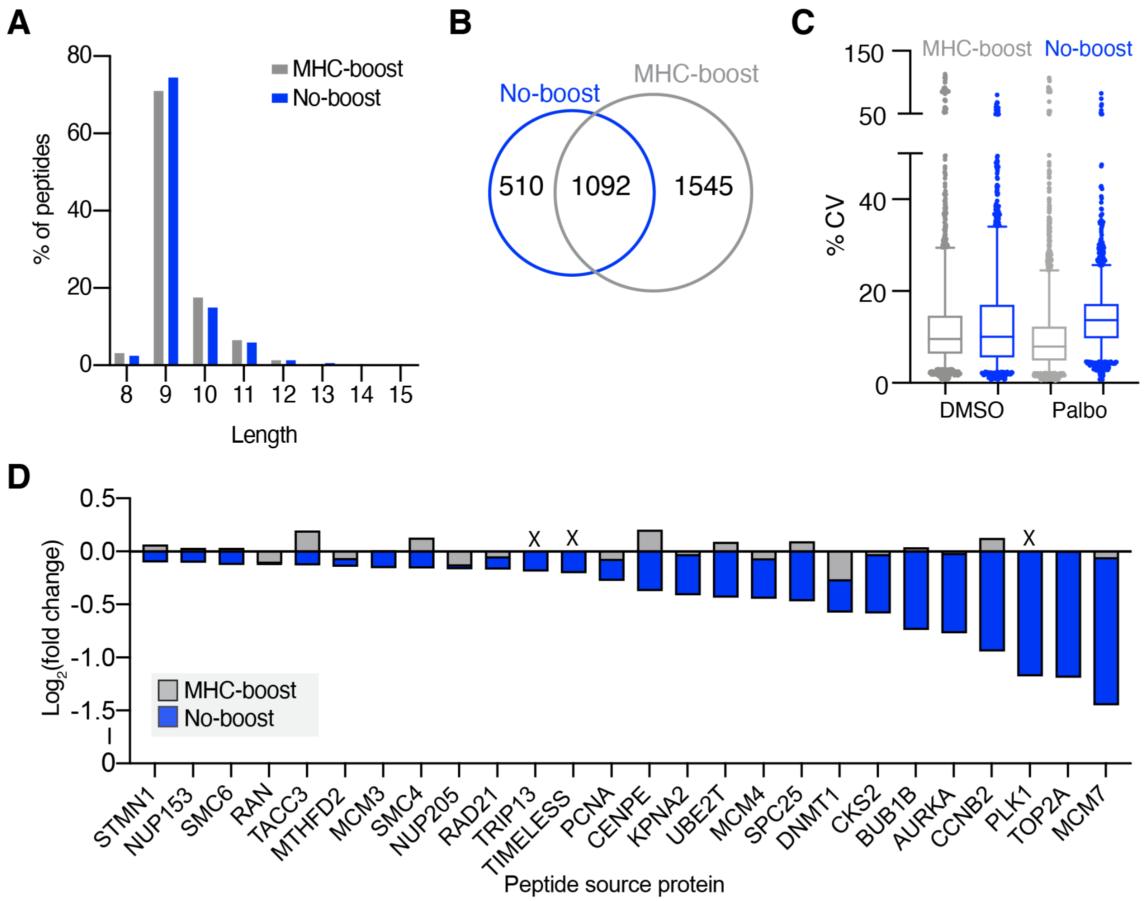

Supplemental Fig. S2.

A, Experimental setup of isobaric labels, cell number, and treatment conditions.

B, Length distribution of pMHCs.

C, Venn diagram of unique pMHC identified in the no-boost (blue) and pMHC-boost (grey) analysis.

D, Coefficients of variation of pMHC-boost and no-boost analyses. Boxes outline the interquartile range, and whiskers the 5 and 95th percentiles. pMHC-boost median CV: DMSO= 9.27%, palbociclib: 7.56%, no-boost DMSO = 9.72%, palbociclib = 13.39%.

E, Change in expression for E2F target pMHCs, plotted by source protein, with palbociclib treatment for the no-boost analysis and corresponding expression levels for the pMHC-boost analysis. X denotes pMHCs which were not quantified in the pMHC-boost analysis.

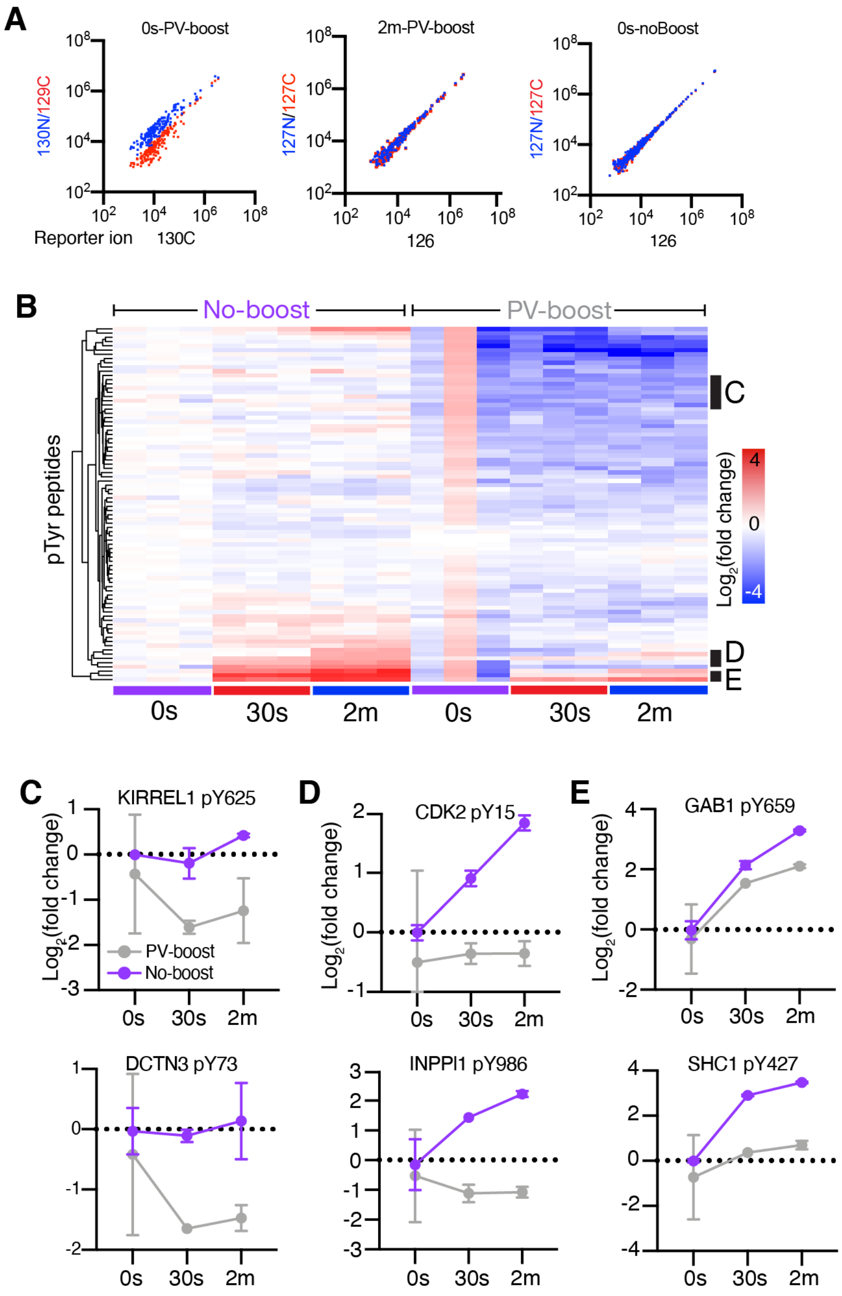

Supplemental Fig. S3.

A, Correlation plots of reporter ion intensities in between 0-s samples in the PV-boost analysis (left, impacted by TMT isotope interference), 2-m PV-boost samples (middle, not impacted by isotopic interference), and 0-s in the no boost analysis as a control (right). Correlation coefficients are ≥ 0.95 for all conditions.

B, Hierarchical clustering (Euclidean) of Log2(fold change) values of pTyr sites identified in both the PV-boost and no-boost analyses. Values are normalized to the mean 0s intensity.

C-E, Selected peptides from corresponding clusters highlighted in A represented as Log_2(fold change) values over mean 0s intensities. Error bars represent +/- standard deviation.

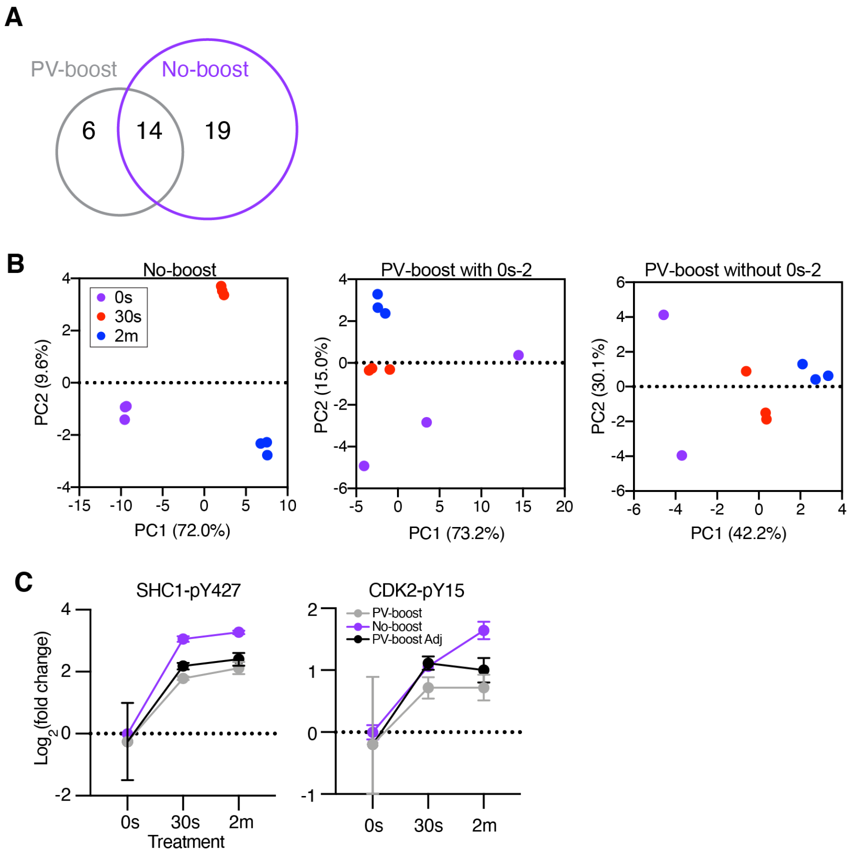

Supplemental Fig. S4.

A, Venn diagram of the number of unique pTyr peptides significantly different (two-tailed T test, $p<0.05$) between the 30s and 2m condition.

B, Samples plotted by principal component 1 (PC1) and PC2 score for no-boost and PV boost analysis including/excluding the 0s-2 sample and colored by EGF stimulation condition. Percentages define the variance described by the plotted PC.

C, Selected peptides represented as Log_2(fold change) values over mean 0s intensities for PV-boost (grey), no-boost (purple), and PV-boost peptides normalized to phosphopeptides with unchanging pTyr signal (average Log_2(fold change) between -0.1 and 0.1 in 30s and 2m conditions) in the no-boost analysis (PV-boost Adj, black). Error bars represent +/- standard deviation.

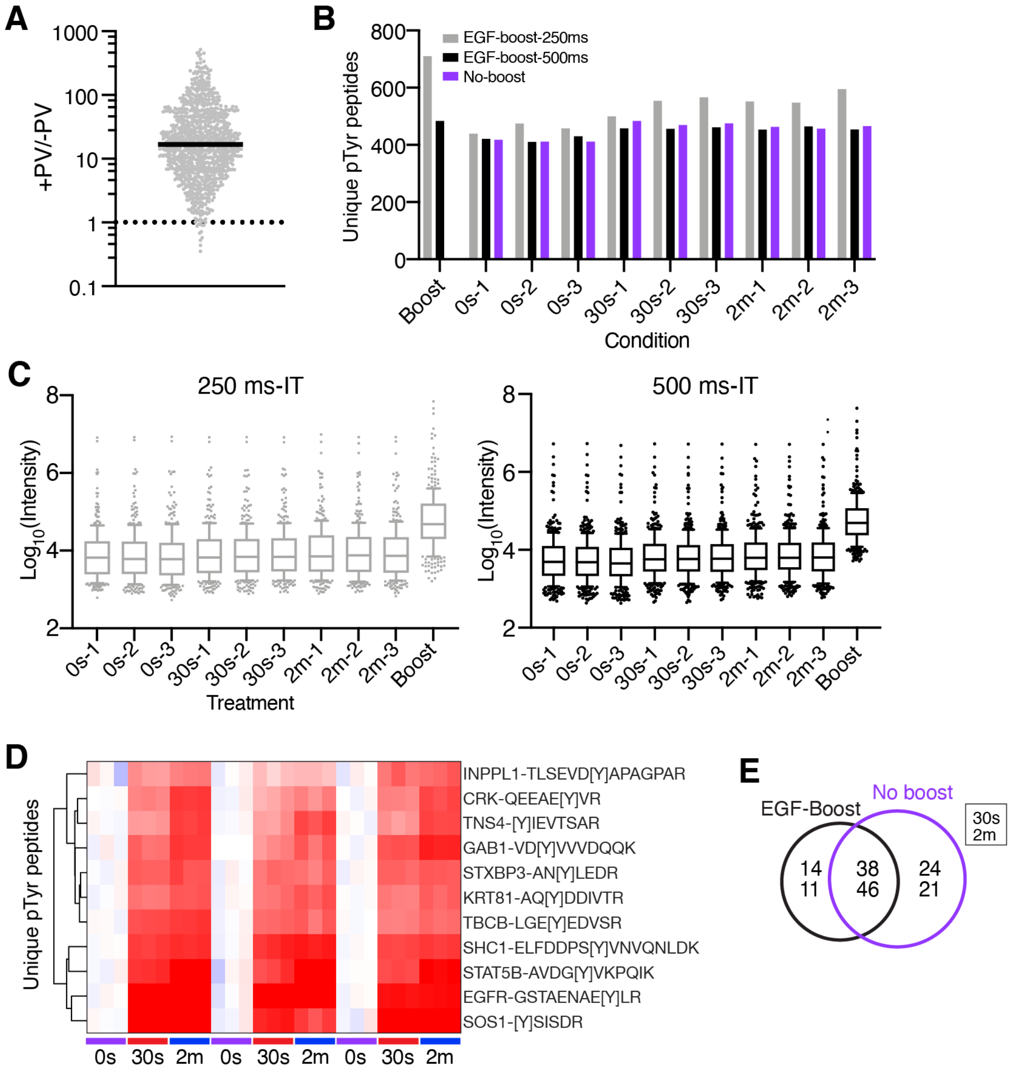

Supplemental Fig. S5.

A, Reporter ion intensity ratios of the median signal of n=3 1 mg Jurkat cell replicates without stimulation and n=2 1 mg jurkat replicates with PV stimulation, as reported by Chua et al., *MCP*, 2020.

B, Number of unique pTyr peptides quantified for each condition for each analysis.

C, Reporter ion intensities for EGF-boost 250 ms IT and 500 ms IT analyses. Boxes outline the interquartile range, and whiskers the 10 and 90th percentiles.

D, Hierarchical clustering of peptides highlighted by black bar in Figure 5A.

E, Venn diagram of number of pTyr peptides significantly different (two-tailed t-test, $p<0.05$) between the 0s and 30s (upper)/2m (lower) conditions using the EGF-boost 500ms and no-boost data.

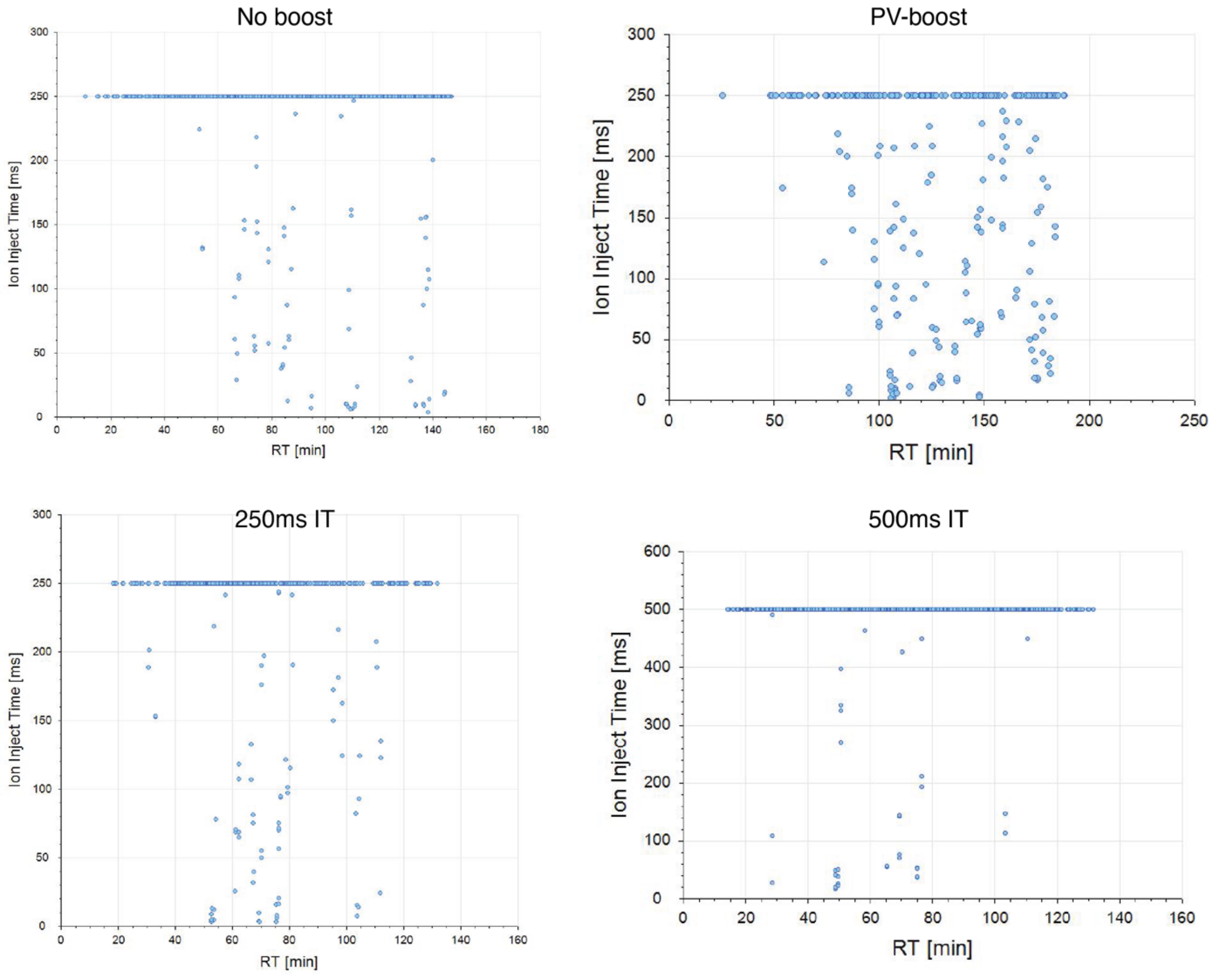

Supplemental Fig. S6.
Injection times for filtered PSMs (blue circles) across analyses. Maximum IT is 250 ms for all analyses, except for the 500 ms IT analysis.

SUPPLEMENTAL TABLES

Supplemental Table S1. Analyzed MS datasets for MHC analyses described in Figures 1-2.

Supplemental Table S2. Filtered search results (PSMs and peptides) for pTyr analyses described in Figures 3-5.

Supplemental Table S3. Source data for the hipMHC quantification in Figure 1G.

Supplemental Table S4. Matrix of values used for the heatmap in Figure 3G.

Supplemental Table S5. Matrix of values used for the heatmap in Figure 5A.
